# Supplementary material for: Characterisation of Aberrant Metabolic Pathways in Hepatoblastoma Using Liquid Chromatography and Tandem Mass Spectrometry (LC-MS/MS)
Source: Cancers (Basel). 2023 Oct 28;15(21):5182. doi: 10.3390/cancers15215182 (PMC10648437; doi:10.3390/cancers15215182)

Chromatograms for the paper submitted to MDPI cancers

Characterisation of aberrant metabolic pathways in hepatoblastoma using liquid chromatography and tandem mass spectrometry (LC-MS/MS)

Alison Whitby*, Pardeep Pabla*, Bhoomi Shastri, Laudina Amugi, Álvaro Del Río-Álvarez, Dong-Hyun Kim, Laura Royo, Carolina Armengol, and Madhumita Dandapani.

Total Ion Chromatograms (posneg)

Untargeted metabolomics (ZIC-pHILIC 15 minute method)

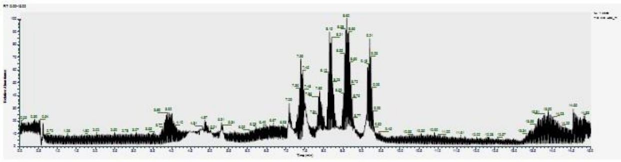

Targeted metabolomics (ZIC-pHILIC 24 minute method)

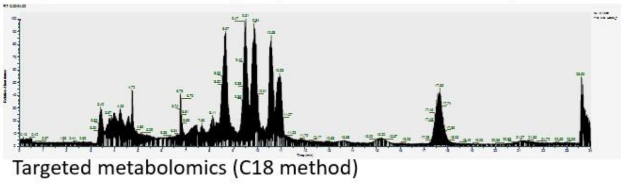

Targeted metabolomics (C18 method)

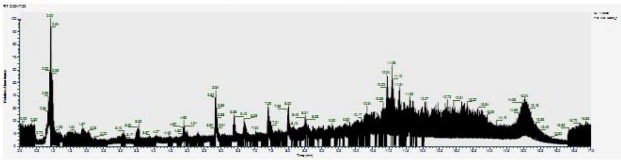

Targeted metabolomics chromatograms of statistically significant metabolites

HILIC

Carnitine

Top: Metabolite in sample HB06_T
Bottom: Internal standard (IS) in HB06_T

MS/MS of metabolite in HB06_T

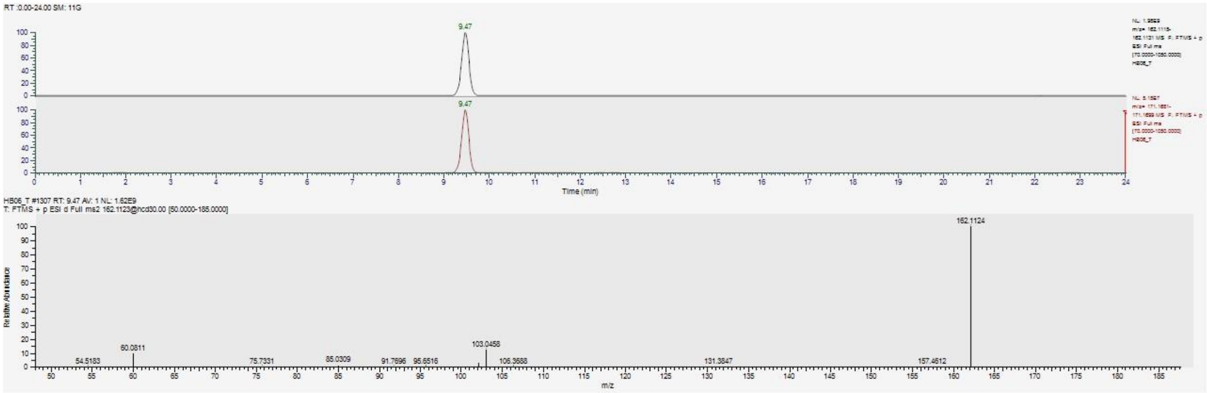

Acetylcarnitine

Top: Metabolite in sample HB06_T
Bottom: Internal standard (IS) in HB06_T

MS/MS of metabolite in HB06_T

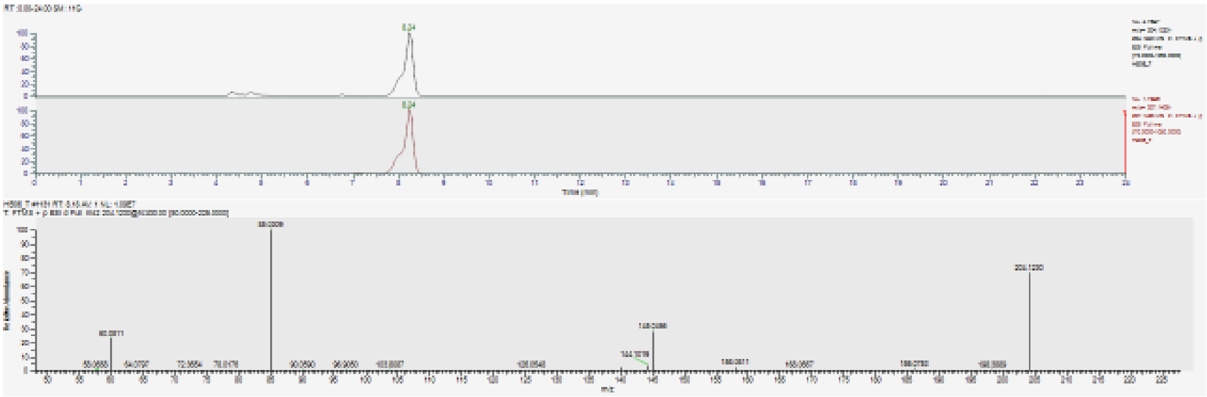

Hydroxybutyrylcarnitine
(using the acetylcarnitine IS for quantification)

Top: Metabolite in sample HB06_T
Bottom: Internal standard (IS) of acetylcarnitine in HB06_T

MS/MS of metabolite in HB06_T

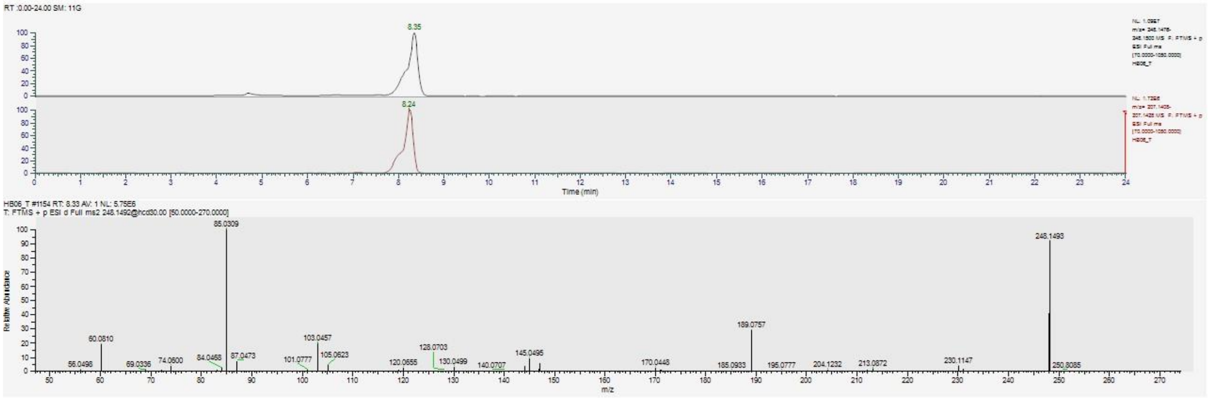

Leucine at 8.43 min, isoleucine at 8.73 min

Top: Metabolites in sample HB06_T
Bottom: Internal standards (IS) in HB06_T

MS/MS of metabolite in HB06_T

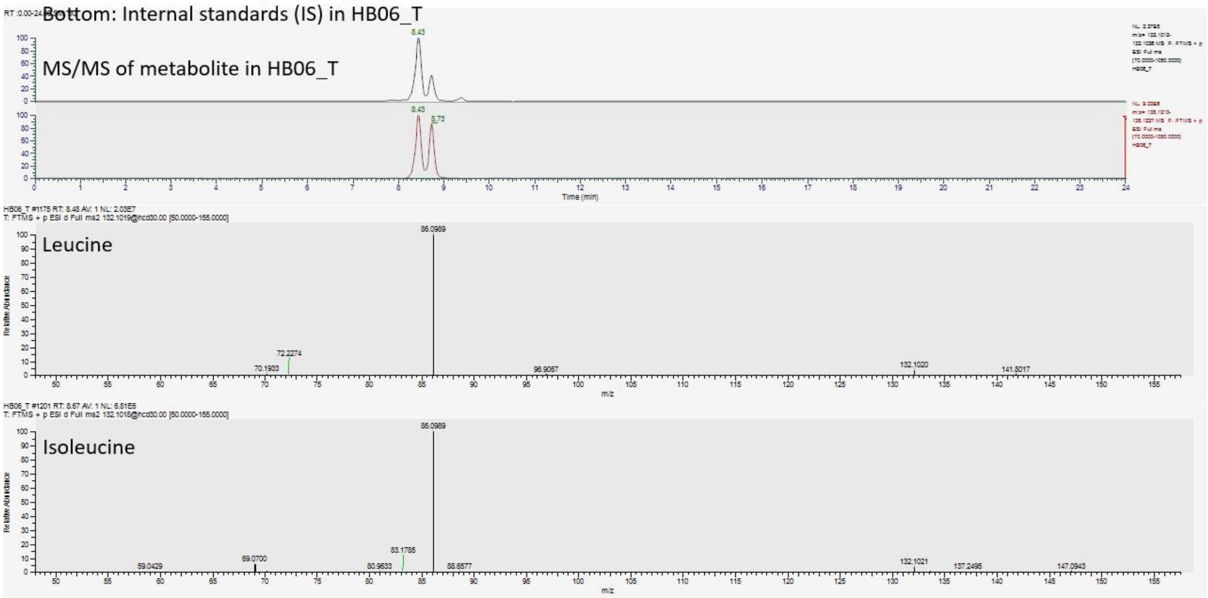

Valine

Top: Metabolite in sample HB06_T
Middle: Internal standard (IS) in HB06_T
Bottom: Standard of valine in calibration curve level 5

MS/MS of metabolite in HB06_T

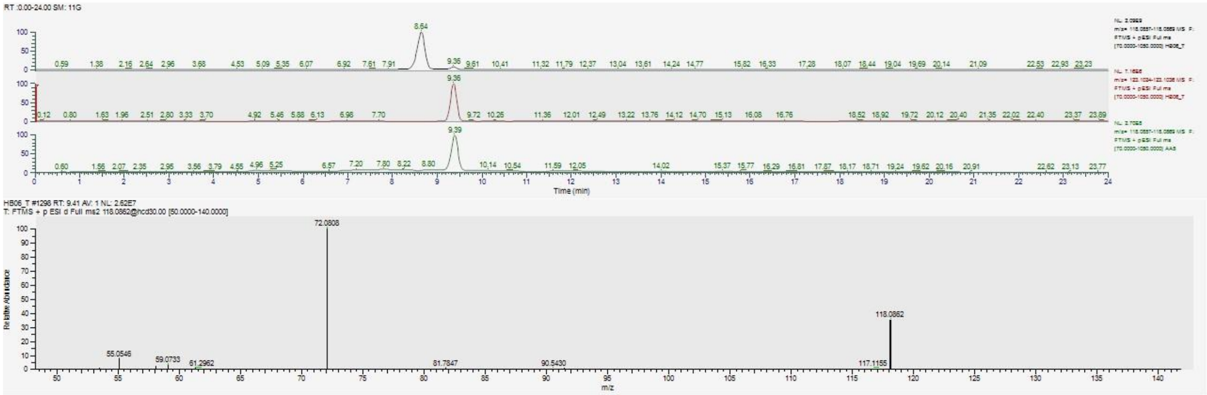

Alanine

Top: Metabolite in sample HB06_T
Bottom: Standard of alanine in calibration curve level 5
(Valine IS was used)

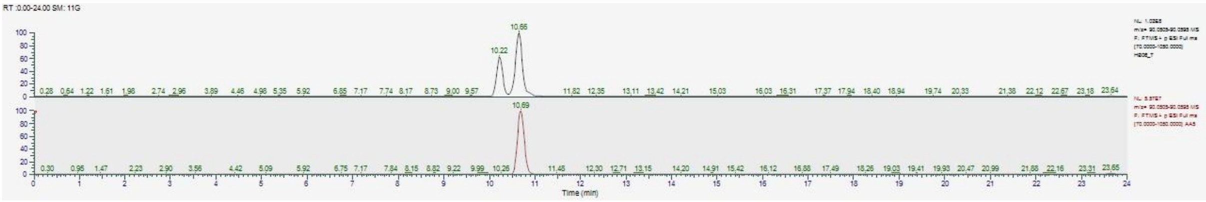

Glycine

Top: Metabolite in sample HB06_T
Bottom: Standard of glycine in calibration curve level 5
(Valine IS was used)

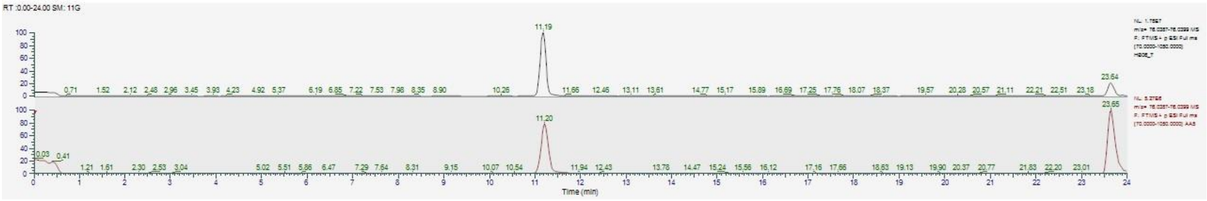

Aspartate

Top: Metabolite in sample HB06_T
Bottom: Standard of aspartate in calibration curve level 5
(Valine IS was used)

MS/MS of metabolite in HB06_T

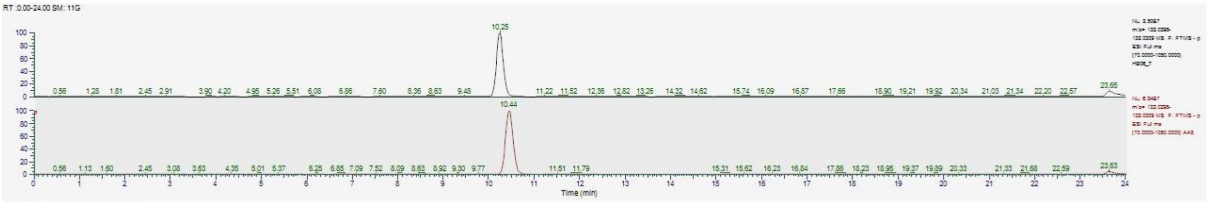

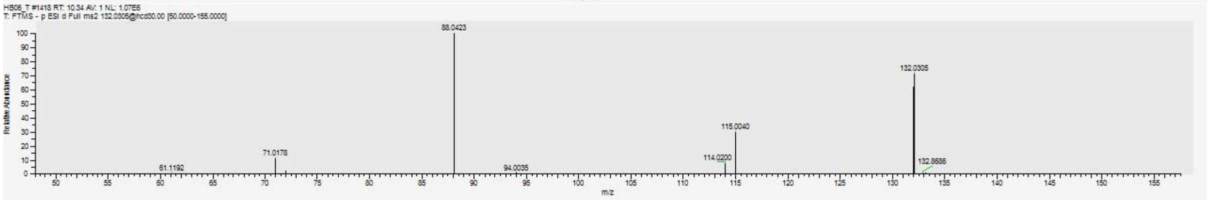

C18

Isobutyrylcarnitine at 4.39 min, butyrylcarnitine (O-butanoylcarnitine) at 4.48 min

Top: Metabolites in sample HB06_T
Bottom: Internal standard (IS) of D3-butyrylcarnitine in HB06_T

MS/MS of butyrylcarnitine metabolite in HB06_T

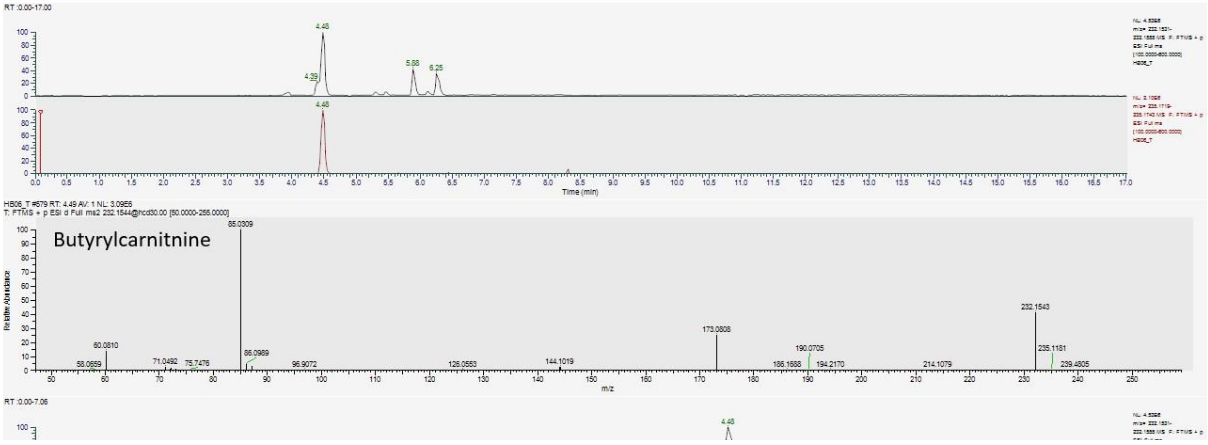

Propionylcarnitine

Top: Metabolite in sample HB06_T
Bottom: Internal standard (IS) in HB06_T

MS/MS of metabolite in HB06_T

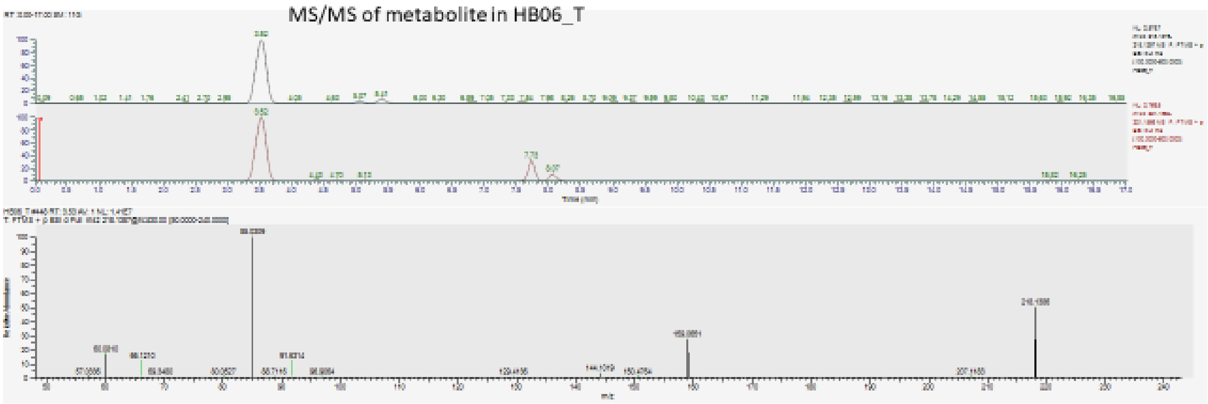

2-Methylbutyroylcarnitine at 5.27 min, isovalerylcarnitine at 5.47 min

Top: Total of isovalerylcarnitine and 2-methylbutyroylcarnitine metabolites as entire smoothed peak in sample HB06_T

Middle: Metabolites in sample HB06_T

Bottom: Internal standard (IS) of D9-C5-Isovalerylcarnitine in HB06_T

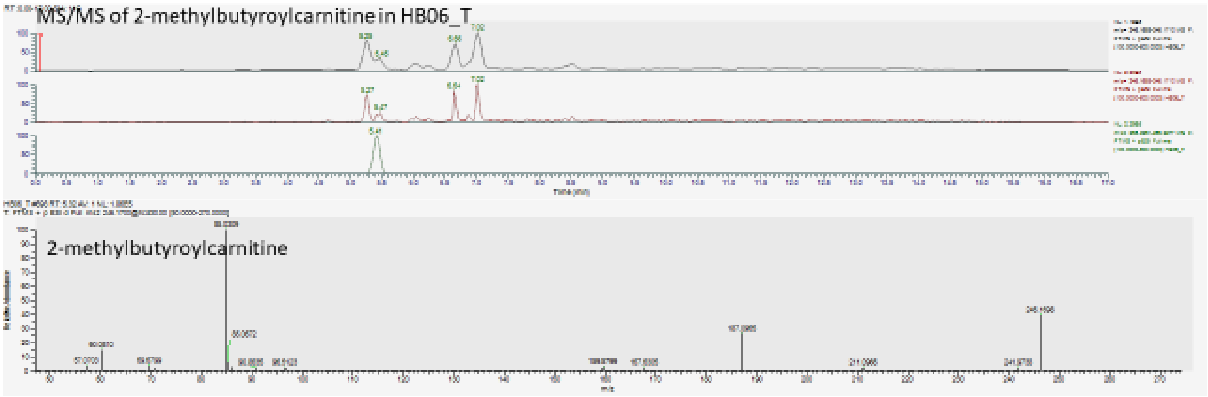

Supplement: Supplementary file 1 [file cancers-15-05182-s001.zip › Chromatograms related to the paper Characterisation of aberrant metabolic pathways in hepatoblastoma using LC-MSMS.pdf]
