# Supplementary material for: Characterisation of Aberrant Metabolic Pathways in Hepatoblastoma Using Liquid Chromatography and Tandem Mass Spectrometry (LC-MS/MS)
Source: Cancers (Basel). 2023 Oct 28;15(21):5182. doi: 10.3390/cancers15215182 (PMC10648437; doi:10.3390/cancers15215182)
Supplement: Supplementary file 1 [file cancers-15-05182-s001.zip › Supplementary information.pdf]

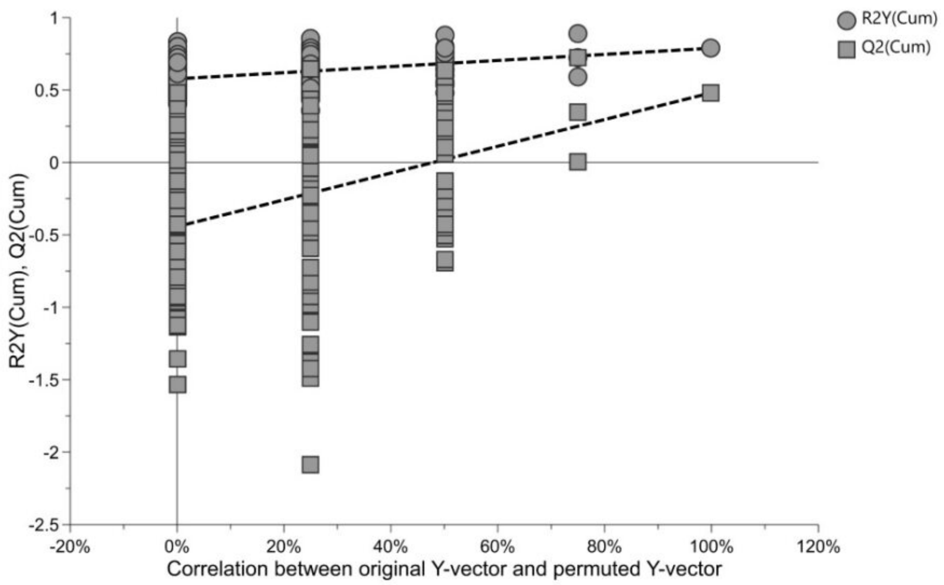

Figure S1: Permutation test of OPLS-DA model shown in Figure 1b.

a)

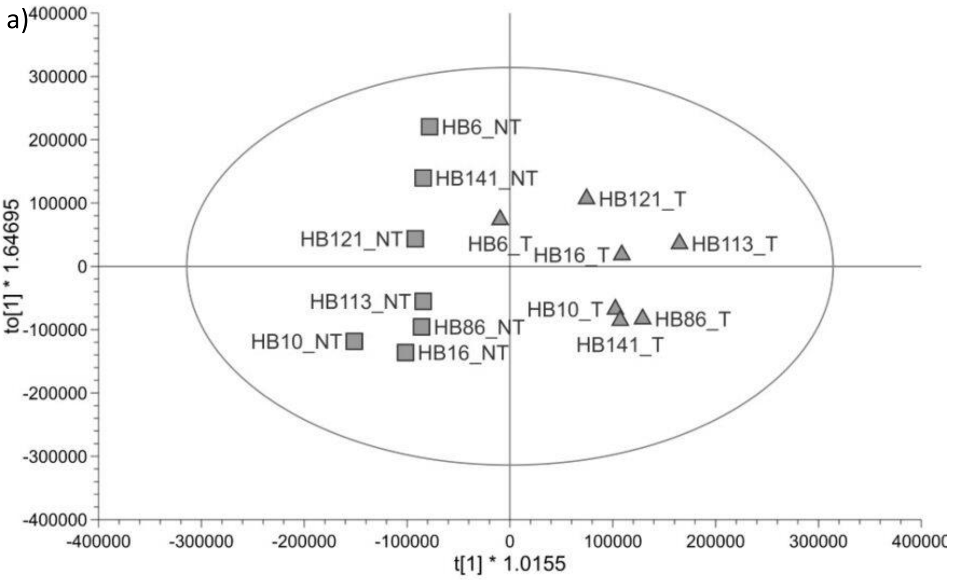

b)

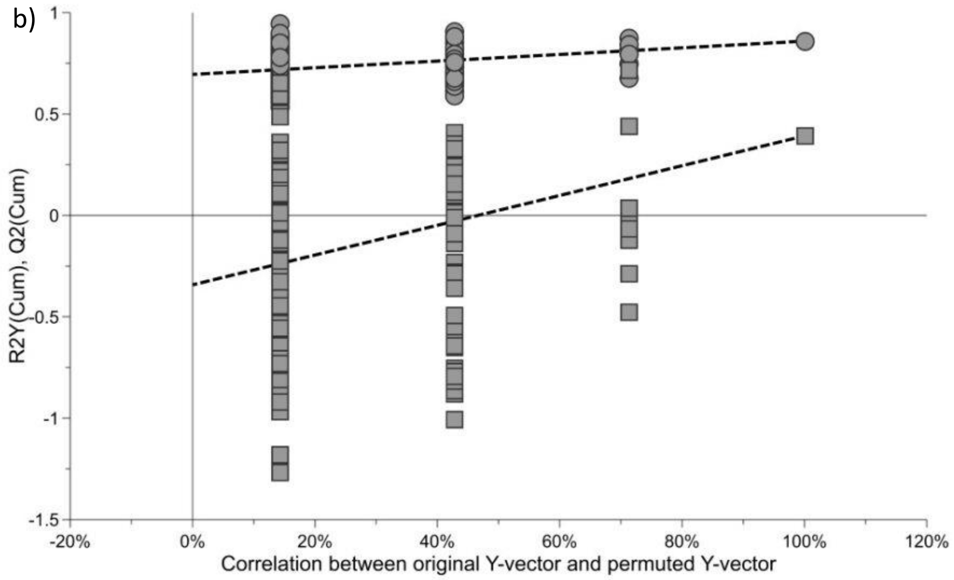

Figure S2: a) OPLS-DA scores plot showing hepatoblastoma ▲ and healthy liver ■ paired samples from paediatric patients omitting patient HB83 (R^2Y 0.859, Q^2 0.392). b) Permutation test of the model.

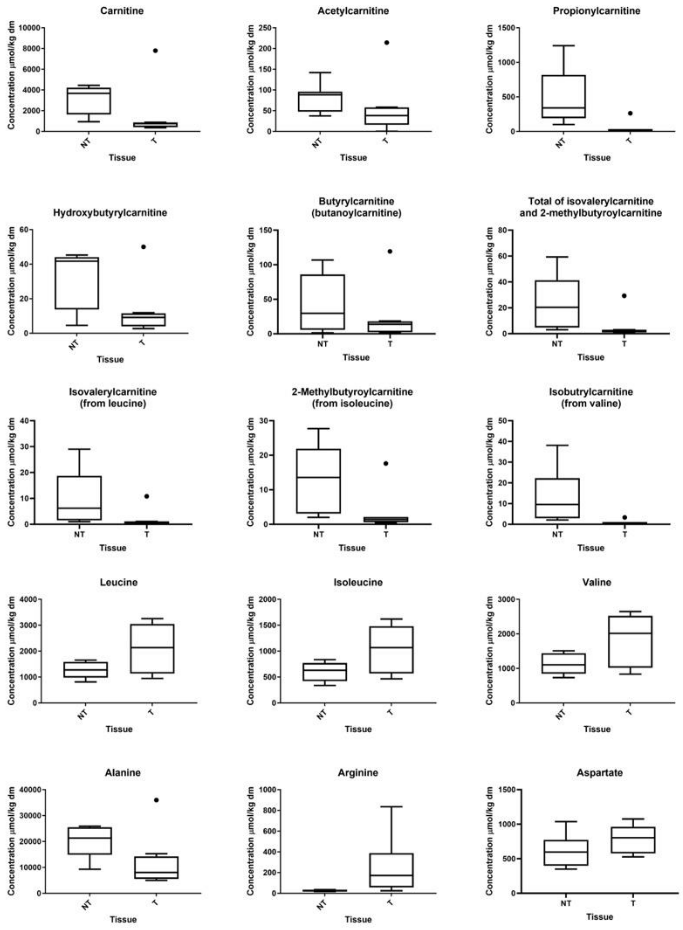

Figure S3: Box and whisker plots with Tukey's whiskers showing HB83_T is an outlier in targeted metabolomics in the case of multiple metabolites, thereby allowing us to remove patient HB83 from our analysis.

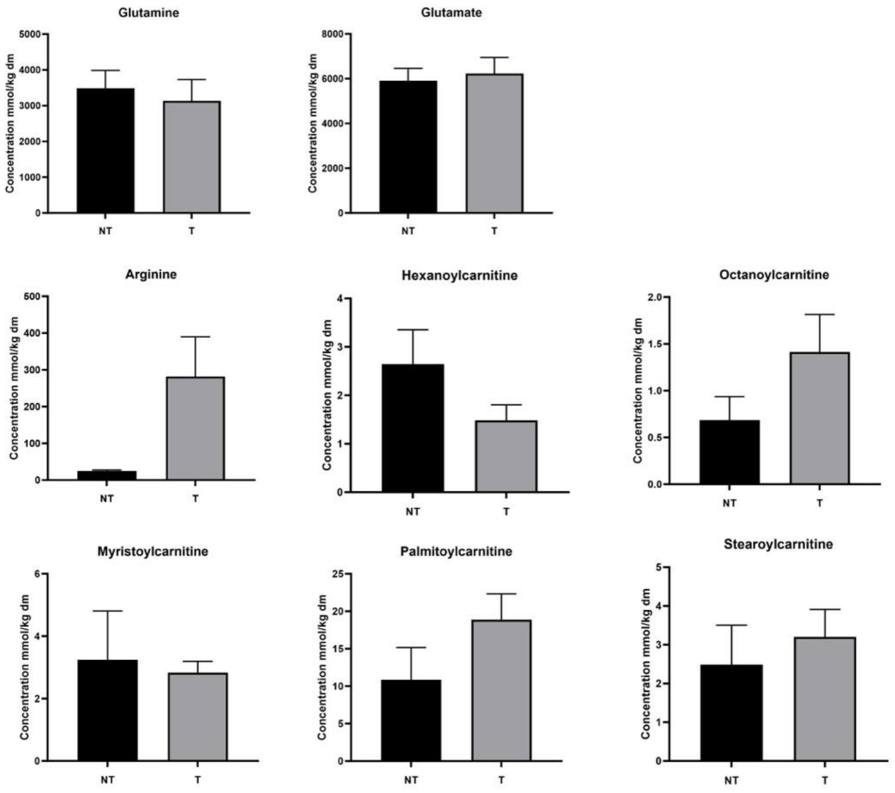

Figure S4: Amino acids and acylcarnitines concentrations in hepatoblastoma (T) and paired healthy liver tissue (NT). Values are means ± standard deviation; (n = 7 paired samples) None were significant.

Table S1: Summary of metabolic pathway analysis with Metaboanalyst (www.metaboanalyst.ca) of the statistically significant metabolites identified by untargeted metabolomics.

Pathway Name	Match Status	p	-log(p)	Holm p	FDR	Impact	Details
**Alanine, aspartate and glutamate metabolism**	5/12	0.014506	1.8385	0.85584	0.5812	0.3109	KEGG SMP SMP SMP
**Nitrogen metabolism**	2/2	0.019702	1.7055	1	0.5812	0	KEGG SMP

**D-Glutamine and D-glutamate metabolism**	2/3	0.05401	1.2675	1	0.8324	0.5	KEGG SMP
**Glyoxylate and dicarboxylate metabolism**	3/7	0.0608	1.2161	1	0.8324	0.03175	KEGG
**Butanoate metabolism**	2/4	0.098759	1.0054	1	0.8324	0	KEGG SMP
**Glutathione metabolism**	2/4	0.098759	1.0054	1	0.8324	0.02675	KEGG SMP
**Citrate cycle (TCA cycle)**	2/4	0.098759	1.0054	1	0.8324	0.12311	KEGG SMP
**Selenocompound metabolism**	1/1	0.14407	0.84143	1	0.85	0	KEGG SMP
**Ether lipid metabolism**	1/1	0.14407	0.84143	1	0.85	0	KEGG
**Propanoate metabolism**	1/1	0.14407	0.84143	1	0.85	0	KEGG SMP
**Lysine degradation**	2/6	0.20675	0.68456	1	1	0.14554	KEGG SMP
**Pyrimidine metabolism**	2/7	0.26512	0.57655	1	1	0.01584	KEGG SMP

**Arginine biosynthesis**	2/7	0.26512	0.57655	1	1	0.11675	KEGG
**Glycerolipid metabolism**	1/2	0.26843	0.57116	1	1	0.04361	KEGG SMP
**Nicotinate and nicotinamide metabolism**	1/2	0.26843	0.57116	1	1	0.1943	KEGG SMP
**Glycerophospholipid metabolism**	2/8	0.32403	0.48942	1	1	0.12907	KEGG
**Aminoacyl-tRNA biosynthesis**	3/15	0.36987	0.43195	1	1	0	KEGG
**Purine metabolism**	3/15	0.36987	0.43195	1	1	0.01451	KEGG SMP
**Porphyrin and chlorophyll metabolism**	1/3	0.37565	0.42522	1	1	0	KEGG
**Glycolysis / Gluconeogenesis**	1/3	0.37565	0.42522	1	1	2.10E-04	KEGG SMP SMP

Table S2: Table of results from the analysis of metabolic pathways using GSEA (Gene Set Enrichment Analysis) confirmed the dysregulation of these specific pathways (FDR<0.25) in HB as compared to NT tissues

Gene Set	Enrichment Score (ES)	Normalized Enrichment Score (NES)	Nominal p-value	FDR q-value	FWER p-Value
WP_ALANINE_AND_ASPARTATE_METABOLISM	-0.9069347	-1.7982053	0	0	0
KEGG_BETA_ALANINE_METABOLISM	-0.8772274	-1.9910481	0	0	0
KEGG_ALANINE_ASPARTATE_AND_GLUTAMATE_METABOLISM	-0.71576357	-2.0145331	0.001908	0.001908	0.001
GOBP_ALPHA_AMINO_ACID_CATABOLIC_PROCESS	-0.7658984	-2.0092547	0.003883	0.003883	0.002
GOBP_BRANCHED_CHAIN_AMINO_ACID_CATABOLIC_PROCESS	-0.88882774	-1.6891409	0.009921	0.009921	0.005
GOBP_AMINO_ACID_CATABOLIC_PROCESS	-0.78353465	-2.0017087	0.002053	0.002053	0.001
REACTOME_TRANSPORT_OF_FATTY_ACIDS	-0.43739185	-0.9198287	0.564007	0.564007	0.304
GOBP_POSITIVE_REGULATION_OF_FATTY_ACID_TRANSPORT	-0.41594616	-1.3285929	0.108607	0.108607	0.053
GOBP_NEGATIVE_REGULATION_OF_FATTY_ACID_TRANSPORT	-0.75177634	-1.5668706	0.021739	0.021739	0.011
GOBP_FATTY_ACID_TRANSPORT	-0.42737868	-1.7121841	0.006061	0.006061	0.003
KEGG_GLUTATHIONE_METABOLISM	-0.56139565	-1.6576638	0.037422	0.037422	0.018
GOBP_GLUTATHIONE_METABOLIC_PROCESS	-0.6309853	-1.9051076	0.008048	0.008048	0.004
GOBP_GLUTATHIONE_CATABOLIC_PROCESS	0.26168445	0.61140496	0.907975	0.907975	0.444
HALLMARK_GLYCOLYSIS	-0.4162613	-1.517957	0.07	0.07	0.035
REACTOME_GLYCOLYSIS	0.39208227	1.2793045	0.225352	0.225352	0.112

KEGG_GLYCOLYSIS_GLUCONEOGENESIS	-0.6698117	-1.9280565	0.001898	0.001898	0.001
REACTOME_CITRIC_ACID_CYCLE_TCA_CYCLE	-0.75022274	-1.6940933	0.01992	0.01992	0.01
REACTOME_PYRUVATE_METABOLISM_AND_CITRIC_ACID_TCA_CYCLE	-0.6535737	-1.9333789	0.00823	0.00823	0.004
KEGG_CITRATE_CYCLE_TCA_CYCLE	-0.75444055	-1.6557847	0.032692	0.032692	0.017
GOBP_INOSINE_METABOLIC_PROCESS	-0.71015054	-1.2990354	0.184265	0.184265	0.089
KEGG_NICOTINATE_AND_NICOTINAMIDE_METABOLISM	-0.56647474	-1.6130509	0.022133	0.022133	0.011
GOBP_NAD_BIOSYNTHESIS_VIA_NICOTINAMIDE_RIBOSIDE_SALVAGE_PATHWAY	-0.7940612	-1.8006079	0.003831	0.003831	0.002

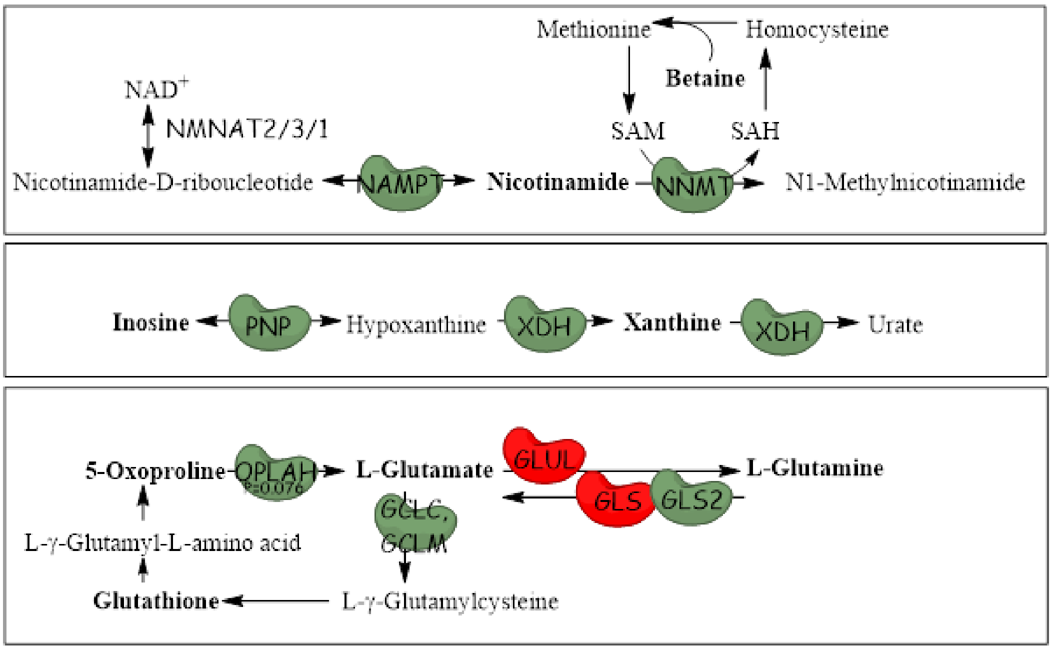

Figure S5: Aberrant metabolic pathways in HB compared to in healthy liver, according to untargeted metabolomics and transcriptomics analyses. Metabolites in bold are significantly decreased in abundance in HB, green genes are significantly decreased and red genes significantly increased in expression in HB.
